# Accuracy of Combined EEG Parameters in Prediction the Depth of Anesthesia

**DOI:** 10.5812/ircmj.1502

**Published:** 2012-12-06

**Authors:** Nourmohammad Arefian, Amir Saied Seddighi, Afsoun Seddighi, Ali Reza Zali

**Affiliations:** 1 Shohada Tajrish Hospital, Shahid Beheshti University of Medical Sciences, Tehran, IR Iran; 2 Shohada Tajrish Hospital, Functional Neurosurgery Research Center of Shohada Tajrish Hospital, Shahid Beheshti University of Medical Sciences, Tehran, IR Iran

**Keywords:** Electroencephalography, Depth, Anesthesia

## Abstract

**Background:**

The importance of proper qualitative evaluation of EEG parameters during surgery has been recognized since many years. Although none of the characteristics based on the frequency, entropy, and Bi spectral characteristics have been regarded as a good predictor for detection of the depth of anesthesia alone. So it seems necessary to study multiple characteristics together.

**Objectives:**

In this study we tried to introduce the best combination of the mentioned characteristics.

**Materials and Methods:**

EEG data of 64 patients undergoing general anesthesia with the same anesthesia protocol (total intravenous anesthesia) were recorded in all anesthetic stages in Shohada Tajrish Hospital. Quantitative EEG characteristics are classified into 4 categories: time, frequency, bi spectral and entropy based characteristics. Their sensitivity, specificity and accuracy in determination of the depth of anesthesia are yielded by comparison with recorded reference signal in awake, light anesthesia, deep anesthesia and brain death patients. Then, with combining 2, 3, 4 and 5 of characteristics and using coded algorithm we determined the error degree and introduced the combination yielding the least error.

**Results:**

Fifteen vectors (of dimension two to five) which yielded the best results were introduced. Vectors combined of entropy based characteristics obtained 100% specificity and sensitivity during all 4 stages.

**Conclusions:**

The combination entropy based characteristics had high accuracy in predicting the depth of anesthesia. Reevaluation of classic indices cortical status index and BIS seems necessary. The next step is to find a system to simplify the evaluation of this information for technicians.

## 1. Background

Evaluation of EEG changes in operating room has special importance in determining the depth of anesthesia. But EEG interpretation is really difficult and needs a high degree of skill (1). Rapid interpretation of EEG needs preparing a system which can alert the anesthesiologist from the depth of anesthesia by using EEG parameters. So, different EEG parameters alone or in combination were used for determining the depth of anesthesia (2). The electroencephalographic (EEG)-derived Bi-Spectral index (BIS) is a sensitive index that reflects the hypnotic component of anesthesia (1, 3). Briefly, the EEG is digitized and processed to detect and remove artifacts. The signal is then analyzed for suppression detection and also fast Fourier-transformation. The suppression is used to compute the burst suppression ratio (BSR). The fast Fourier transform is used to compute a relative beta ratio (Beta Ratio) and also to compute the BI Spectrum, from which the relative synchrony of fast and slow wave (Synch Fast Slow) is derived. All of these components are combined by using multipliers derived from discriminant analysis, with the result scaled from 0 to 100. However, the BIS machine displays only BSR and spectral edge frequency 95% (SEF95), which is not included in the published calculation process of BIS. There is no way to know how Beta Ratio and Synch Fast Slow are changed during anesthesia. The exact algorithm used to synthesize the parameters to BIS values is still not known (4). Four Cortical surface monitoring (CSM) has been introduced by Demeter Company in 2004 which indicates cortical status index (CSI). CSI uses 4 different characteristics in time and frequency of EEG signal as the input of Anfis system. Clinical studies show that there is a great correlation between CSI and BIS (4-7). In this study we tried to study multiple parameters together and choose the best combination for predicting depth of anesthesia. Nowadays, we use two standard indices CSI and BIS, which are combinations of several of the mentioned parameters.

## 2. Objectives

In this study we tried to study multiple parameters together and choose the best combination for predicting the depth of anesthesia.

## 3. Material and methods

**Figure 1:**
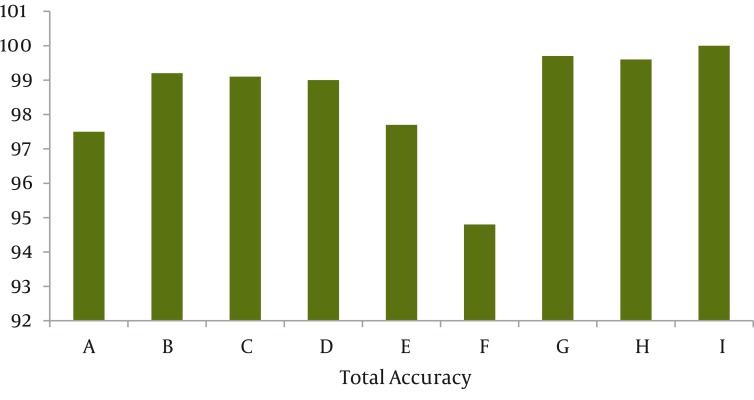
The total Accuracy of EEG Parameters in Prediction of Anesthesia States

We recoded physiologic and anesthetic characteristics, and also their EEG signals, the depth of anesthesia based on CSI index, muscle relaxation degree, hemodynamic parameters such as blood pressure, heart rate and arterial O2 saturation of 64 patients undergoing general anesthesia between April 2005 and March 2006. EEG signals were recorded by CSM and CSI index was also recorded for every patient. The data are summarized in Table 1. We determined muscle relaxation by using EMG information and nerve stimulator (XAVANT). Muscle relaxation degree was calculated with nerve stimulator. EEG was recorded by CSM with 3 superficial electrodes on FPZ position (positive in the middle of forehead), TS (negative on left mastoid) and reference electrode on FP1 (left frontal). For differentiating different stages of anesthesia we described 4 classes of anesthesia: awake, light anesthesia, deep anesthesia and isoelectric state. We recorded 15 minutes for each class, summing overall 60 minutes of recording. The awake class reference data included 15 minutes of EEG, recorded from 3 healthy awake people (5 minutes each). To omit the blinking artifacts we advised them to close their eyes and concentrate on a special subject. The light anesthesia stage was defined from the time of initial drug injection to intubation and from the discontinuation of drugs until full awareness of the patients based on anesthesiologist assessment. EEG of 14 different patients anesthetized with the above protocols were assessed to have a 15 minutes reference signal for this class. The deep anesthesia stage included 15 minutes of EEG signal from the above mentioned 14 patients as recorded in phase 3 of anesthesia. Isoelectric class data are recorded from 3 people with brain death. By using BIS classifier and one of the 14 introduced characteristics (summarized in Table 2) we differentiated the different classes of anesthesia. Then we determined sensitivity, specificity and total accuracy of each of these parameters in 4 classes of anesthesia. The results are shown in Figure 1. Then different collections of characteristics arranged in multidimensional vectors ranging from two to five dimensions were used for classifying the depth of anesthesia. The vectors differ in the number and the type of characteristics they encompass. We explained an error estimated to choose the best combination. Then we tried to minimize this error by using genetic algorithm (based on coding). Among all these characteristics we tried to choose the best multidimensional vector. In generational genetic algorithm procedure:

1-We chose the initial populations of individuals

2-Evaluate the fitness of each individual in each population

2-Repeat on this generation until termination (time limit, sufficient fitness achieved, etc.):

3-Select the best-fit individuals for reproduction

4-Breed new individuals through crossover and mutation operations to give birth to offspring

5-Evaluate the individual fitness of new individuals

6-Replace least-fit population with new individuals

**Table 1:** General Characteristic of the Patients

Types of Surgery	Sex, Male, Female	Age, y	Weight, kg
**Gastrectomy**	M	70	60
**Thyroidectomy**	F	39	80
**Cholesystectomy**	F	74	80
**Umbilical Hernia**	M	67	70
**Thyroidectomy**	M	16	50
U**nilateral Varicoselectomy**	M	31	75
**Unilateral Varicoselectomy**	M	45	70
**B.K Amputation**	M	34	50
**Hernia**	F	50	64
**Z Plasty**	M	22	76
**Umbilical Hernia**	F	46	68
**Umbilical Hernia**	F	65	61
**TURT**	M	66	58
**Hernia**	F	23	96
**TOS**	F	15	50
**Gastrectomy**	F	32	90
**Hernia**	M	40	60
**Gastrectomy**	F	30	51
**TURP**	M	75	70
**Thyroidectomy**	F	69	81
**Thyroidectomy**	M	22	70
**TUL**	M	45	80
**Umbilical Hernia**	M	35	74
**TOS**	F	45	63
**BK Amputation**	M	65	75
**Gastrectomy**	M	64	76
**Hernia**	M	35	80
**Gastrectomy**	F	29	65
**Umbilical Hernia**	M	50	74
**Thyroidectomy**	M	43	82
**Prostatectomy**	M	70	60
**Gastrectomy**	F	39	80
**Inguinal Hernia**	F	74	80
**Umbilical Hernia**	M	67	70
**Umbilical Hernia**	F	16	50
**Cholesystectomy**	M	31	75
**Unilateral Varicoselectomy**	M	45	70
**B.K(LL)Amputation**	M	34	50
**Gastrectomy**	M	50	64
**Z Plasty**	M	22	76
**TUL**	F	46	68
**Thyroidectomy**	F	65	61
**TURT**	M	66	58
**Umbilical Hernia**	F	23	96
**Cholesystectomy**	F	15	50
**TUL**	F	32	90
**Umbilical Hernia**	M	40	60
**Umbilical Hernia**	F	30	51
**TURP**	M	75	70
**Gastrectomy**	F	69	81
**Thyroidectomy**	M	22	70
**TUL**	F	45	80
**TUL**	F	35	74
**TOS**	F	45	63
**AK Amputation**	M	65	75
**Gastrectomy**	M	64	76
**Umbilical Hernia**	M	35	80
**TUL**	F	29	65
**Umbilical Hernia**	F	50	74
**Thyroidectomy**	M	43	82
**TUL**	M	70	60
**AK Amputation**	F	56	80
**TURT**	M	74	80
**Thyroidectomy**	F	67	70

**Table 2:** EEG Characteristic Abbreviations

Abbreviations	Definitions
**SEF**	Spectral Edge Frequency
**MF**	Median Frequency
**DBP**	Delta Band Power
**TBP**	Teta Band Power
**ABP**	Alpha Band Power
**BBP**	Beta Band Power
**ARF**	Alpha Ratio Frequency
**BRF**	Beta Ratio Frequency
**TRF**	Teta Ratio Frequency
**BSFSR**	Bispectral Synch Fast Slow Ratio
**BS%**	Burst Suppression%
**ShE**	Shannon Entropy
**SE**	Spectral Entropy
**RE(-1)**	Renyi Entropy(-1)
**RE(3)**	Renyi Entropy(3)
**SVDE**	Singular Value Decomposition Entropy
**AE**	Approximate Entropy
**L-Z V**	Lempel-Ziv Entropy
**WBC**	Wavelet Based Characteristic

## 4. Results

Our patients included 29 women and 35 men, with the age range 14-78 years old (mean 45.7 year, SD: 18.5), weight range 47-105 kg (mean 70.2 year, SD: 13.3) (Table 1). At first, we used combinations of frequency based characteristics including: spectral edge frequency, median frequency, delta band power, theta band power, alpha band power, beta band power, alpha ratio, beta ratio and theta ratio together with BSR (Burst Suppression Ratio) for classification. By using genetic algorithm to minimize error, we understood that among 2 dimensional vectors the one consisting of β- frequency coefficient and β- frequency band power had the best result. (Total accuracy: 97.5%). By using 3 characteristics, the vector including median frequency, spectral edge frequency and BSR had the greatest accuracy in classification (99.17 %). Three mentioned characteristics plus α band power were the best quadruple vectors and by adding α frequency coefficient to them the resulted pentacle vector had the greatest accuracy. By using CSI index (Alpha Ratio, Beta Ratio, Theta Ratio and BSR) we gained 97.67% accuracy in classifying 4 stages of awake, light, deep and isoelectric states. The results of BIS score were a little worse. The accuracy decreased to 94.79% in this situation. Approximate entropy characteristic associated with Shannon entropy can differentiate different classes of anesthesia with 99.67% accuracy. These two factors plus (3) Reyna entropy were three characteristics that had better predictability by forming a triple vector, although our accuracy decreased to 99.58% in comparison with double vectors.

**Figure 2:**
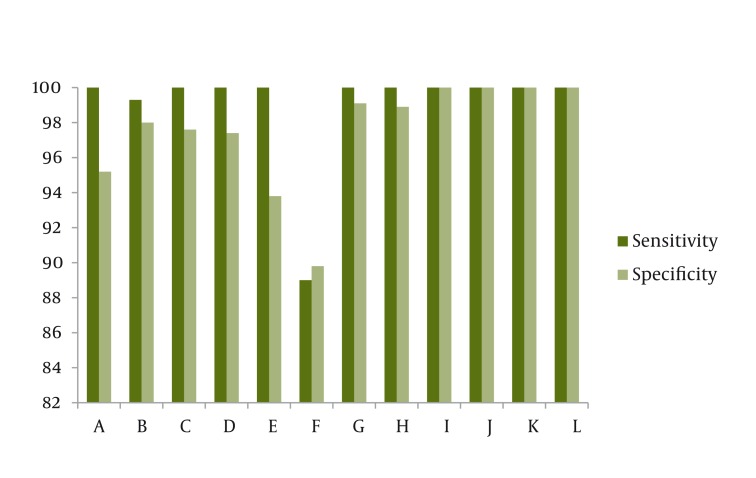
The Sensitivity and Specificity of the Accuracy of Each of the Parameters in the Deep Anesthesia State

By using 4 or 5 characteristics among 7 entropy based characteristics we gained 100% accuracy. By using approximate entropy, (3) Reyna entropy, spectral entropy and Lempel- ZIV complex as a 4 dimensional characteristic vector from EEG signal, the accuracy was 100%.These 4 characteristics plus Shannon characteristic created the best resolution power among pentacle characteristic vectors. Approximate entropy plus delta band power had the greatest ability in differentiation of the different stages of anesthesia (99.92%) by increasing our characteristics to 19. The approximate entropy, Shannon entropy, BS percent had the best result (100% accuracy) in differentiation of the different stages of anesthesia by taking test signals to 3 dimensional characteristic space. Data are shown in Figure 2.

## 5. Discussion

Over the years, many methods have been proposed to quantify the depth of anesthesia from an EEG signal. Most of these methods quantify, by power spectral analysis, the increase in the low-frequency components of the EEG signal that occurs as the anesthetic concentration increases. Calculation of SEF95 (spectral edge frequency 95%: the frequency below which 95% of the power in the spectrum resides), is one of these methods. It has been shown that SEF95 may predict the depth of anesthesia fairly well. However, the SEF response to chang the depth of anesthesia is biphasic (8, 9). At surgical levels of anesthesia, Synch Fast Slow may be an important component of BIS.1 The core technology for calculating BIS is Bispectral analysis (1, 6, 10). BSR is a time-domain EEG parameter that was developed to quantify burst suppression. Similar to our results, Bruhn reported that BSR > 40% was linearly correlated with BIS values in the range of 30 to 0 (11). Finally it’s clear that BIS will not reach to its primary basic level only after patient recovery and minimal EEG signal can end to controversial results. In our study, BIS triple characteristic vector had 94.8% accuracy which was the least accuracy among other characteristic vectors. BIS index had low accuracy in light and deep anesthesia but 100% accuracy, sensitivity and specificity in awareness & ISO electric stage which could be the result of using β coefficient characteristic and BSR in this index. CSI uses 4 different characteristic of EEG signal frequency and time as the input of ANFIS system. Many clinical studies have revealed that CSI and BIS have close relationship. BIS and CSI indices have good relationship (93%, 92% respectively) with clinical standards of depth of anesthesia such as OAAS (12). The sensitivity, specificity and accuracy of CSI are 100% in awake and isoelectric stage such as BIS but its pitfall is in differentiation of light anesthesia. Total accuracy of quadruple CSI criteria is less than other vectors in Figure 2 except double vector of β coefficient, θ band power and the triple vector of BIS. In general, vectors consisting of entropy based characteristics had the best results such as the triple vector of approximate entropy, Shannon entropy and BSR and pentaple vector of all entropies (3) Reyna entropy, Lempel- ZIV, Shannon, approximate and spectral entropy) and quadruple vector entropy (approximate, Lempel- ZIV, (3) Reyna and spectral entropy) had 100% sensitivity, specificity and total accuracy in all 4 stages of anesthesia. The parameters are shown in Figure 2, shows the advantages of CSI and BIS. By evaluating the effective parameters in determining the depth of anesthesia, maybe it’s the time to introduce them to specialist. Also automatization of EEG interpretation may need useful systems for practicing these indices.
